# Influence of Androgen Receptor on the Prognosis of Breast Cancer

**DOI:** 10.3390/jcm9041083

**Published:** 2020-04-10

**Authors:** Ki-Tae Hwang, Young A Kim, Jongjin Kim, Jeong Hwan Park, In Sil Choi, Kyu Ri Hwang, Young Jun Chai, Jin Hyun Park

**Affiliations:** 1 Department of Surgery, Seoul Metropolitan Government Seoul National University Boramae Medical Center, 39, Boramae-Gil, Dongjak-gu, Seoul 156-707, Korea; michael5@hanmail.net (J.K.); kevinjoon@naver.com (Y.J.C.); 2 Department of Pathology, Seoul Metropolitan Government Seoul National University Boramae Medical Center, Seoul 156-707, Korea; pathgirl@daum.net (Y.A.K.); hopemd@hanmail.net (J.H.P.); 3 Department of Internal Medicine, Seoul Metropolitan Government Seoul National University Boramae Medical Center, Seoul 156-707, Korea; hmoischoi@hanmail.net (I.S.C.); jinhyunpak@gmail.com (J.H.P.); 4 Department of Obstetrics & Gynecology, Seoul Metropolitan Government Seoul National University Boramae Medical Center, Seoul 156-707, Korea; orangemd@snu.ac.kr

**Keywords:** androgen receptor, breast neoplasms, disease-free survival, overall survival, prognosis

## Abstract

We investigated the prognostic influence of androgen receptor (AR) on breast cancer. AR status was assessed using immunohistochemistry with tissue microarrays from 395 operable primary breast cancer patients who received curative surgery. The Kaplan–Meier estimator was used to analyze the survival rates and a log-rank test was used to determine the significance of the differences in survival. The Cox proportional hazards model was used to calculate the hazard ratio (HR) and the 95% confidence interval (CI) of survival. There were 203 (51.4%) subjects with a low expression of AR, and 192 patients (48.6%) with a high expression rate. The high AR expression group showed superior overall survival (*p* = 0.047) and disease-free survival (*p* = 0.004) when compared with the low AR expression group. The high AR expression group showed superior systemic recurrence-free survival when compared with the low AR expression group (*p* = 0.027). AR was an independent prognostic factor for both overall survival (HR, 0.586; 95% CI, 0.381–0.901; *p* = 0.015) and disease-free survival (HR, 0.430; 95% CI, 0.274–0.674; *p* < 0.001). A high AR expression was a significant favorable prognostic factor only in the subgroups with positive hormone receptors (HRc) and negative human epidermal growth factor receptor 2 (HER2) when considering disease-free survival (*p* = 0.026). The high AR expression group was significantly associated with superior overall survival and disease-free survival when compared with the low AR expression group with breast cancer patients. AR was a significant independent prognostic factor for both overall survival and disease-free survival. The prognostic impact of AR was valid in the HRc(+)/HER2(−) subtype when considering disease-free survival. These findings suggest the clinical usefulness of AR as a prognostic marker of breast cancer in clinical settings.

## 1. Introduction

The androgen receptor (AR) and its related pathways are emerging as a therapeutic target in breast cancer, and the possibility of AR-targeted therapy in breast cancer is under active investigation [1]. AR expression is frequently observed in breast cancer and the rate of AR positivity is reported to be 60%–80% [2]. AR positivity rate ranges widely, mainly depending on the study population and definition of AR positivity in each study [3,4]. AR expression rate is also reported to be different across breast cancer subtypes [5,6,7]. AR expression and its signaling pathway have been reported to have an important role in the pathogenesis and progression of breast cancer [8,9]. Of note, the biologic effect of AR on breast cancer has been reported to be different according to the estrogen receptor (ER) status. AR is proposed to have an inhibitory effect on cancer cells via inhibition of ER activity in ER-positive breast cancers [8,10], while AR is suggested to promote the proliferation of cancer cells in ER-negative breast cancers [6,11,12].

AR expression is reported to be closely related to clinicopathologic parameters of breast cancer and also related to the prognosis of patients to breast cancer. Efforts have been made to reveal the prognostic role of AR in breast cancer, but the results have been controversial to date. Some studies have reported a favorable prognostic effect of AR [3,4,5,13,14,15,16], but others have reported no association between AR expression and breast cancer prognosis [2,12,17]. Furthermore, other studies have reported an adverse prognostic impact of AR positivity [6,18]. This inconsistency in the research for the prognostic role of AR is greater when it is analyzed by subgroups of various clinicopathologic parameters. The prognostic impact of AR has been reported to be closely related to the ER, the progesterone receptor (PR), and the human epidermal growth factor receptor 2 (HER2) in breast cancer [3,5,15,19,20]. Accordingly, breast cancer subtype has also been reported to be a crucial factor in terms of AR prognostication [5,6,7,12]. Further studies are needed to validate the prognostic role of AR in breast cancer. 

These inconsistencies in the literature regarding the prognostic role of AR in breast cancer precipitated this study. In this study, we investigated the prognostic role of AR in breast cancer patients and looked at both overall survival and disease-free survival. We also investigated the association of AR status with various clinicopathologic features including breast cancer subtypes.

## 2. Materials and Methods

### 2.1. Study Subjects

Patients with primary invasive breast cancer were prospectively registered in the Boramae Hospital Breast Cancer Registry. In 2012, we made tissue microarrays using cancer tissues from 420 registered patients and designed this study to analyze the data. Three patients were excluded because of duplication. Eleven patients, who were initially diagnosed as having stage IV breast cancer, were excluded. An additional 11 patients were excluded because there was an insufficient amount of remnant tumor tissues for AR staining. Finally, 395 patients were enrolled in this study. Data acquisition and analysis were performed in compliance with protocols approved by the institutional review boards of the Seoul Metropolitan Government Seoul National University Boramae Medical Center (approval numbers: 16-2016-140, 16-2017-57). Written informed consent was obtained from all participants prior to the study.

### 2.2. Preparation of Tissue Microarray and Immunohistochemistry of AR

The tissue microarrays were constructed with 2 mm diameter cores of formalin-fixed paraffin-embedded tissue blocks from representative tumor areas. Immunohistochemical staining was performed on the tissue microarrays with the DAKO Omnis autostainer (DAKO-Agilent Technologies, Santa Clara, CA, USA) using a rabbit anti-AR (SP107) monoclonal antibody (1:100, Cell Marque, Rocklin, CA, USA). The results of the staining were evaluated based on the proportion of stained tumor cells and the average intensity of the nuclear staining in the tumor cells. The proportion scores were dichotomously classified as follows: ≤10% of tumor cells vs. >10% of tumor cells. The intensity scores were scored on the following scale: 0, negative; 1, weak; 2, moderate; 3, strong (Figure S1). An Allred score was calculated from the sum of the proportion score and the intensity score according to previous reports [21,22].

### 2.3. Definition of Variables

AR status was classified as low vs. high using the intensity score with a cut-off value of 10% with immunohistochemistry. The stage was described according to the 8th edition of the American Joint Committee on Cancer. The status of the ER or the PR was interpreted as positive if there were at least 1% positive tumor nuclei in the sample. A sample is considered negative if <1% or 0% of tumor cell nuclei are immunoreactive [22,23]. The hormone receptor (HRc) status was defined as positive when the immunohistochemical test for either ER or PR was positive. The HER2 status was defined as positive or negative based on the results of the immunohistochemical test and an in situ hybridization test [24]. Antibodies for immunohistochemistry were all in the ready-to-use format and immunohistochemical stains were performed using Benchmark Ultra automated slide stainer (Roche Diagnostics, Indianapolis, IN, USA) according to manufacturer’s instruction. Immunohistochemical staining for ER (monoclonal rabbit anti-human ER, Roche Diagnostics, clone SP1, reference 790-4324), PR (monoclonal rabbit anti-human PR, Roche Diagnostics, clone 1E2, reference 790-2223), and HER2 (monoclonal rabbit anti-human HER2, Roche Diagnostics, clone 4B5, reference 790-4493) had been performed using whole tumor section at the time of diagnosis, and the staining results were utilized for this study. Human endometrial tissue was used as positive control for ER and PR stains. Breast cancer tissue previously diagnosed as HER2 positive was used as positive control for HER2 staining. Positive control tissues were processed in the same manner as the patient specimens. Instead of primary antibody, slides stained with CONFIRM negative control rabbit Ig (Roche Diagnostics, reference 790-1029) were used as negative control. The histologic grade was determined with the modified Scarff-Bloom-Richardson grading system. The body mass index was defined as the ratio of body weight in kilograms to height in square meters. Finally, the breast cancer subtypes were classified into four groups according to HRc and HER2 as follows: HRc(+)/HER2(−), HRc(+)/HER2(+), HRc(−)/HER2(+), and HRc(−)/HER2(+).

### 2.4. Statistical Analyses

A two-sample *t*-test was used to determine the statistical difference of the mean ages, and a Pearson’s χ^2^ test was used to determine the statistical difference of all of the other baseline characteristics. Recurrence was defined as any first event of local, regional, systemic, and contralateral breast cancer recurrence. The time duration of overall survival was calculated as the time from the initial diagnosis until death from any cause. The time duration for disease-free survival was defined as the time difference from the operation until any recurrence. The Kaplan–Meier estimator was used to analyze the survival rates and a log-rank test was used to determine the significance of the difference between the survival curves. The Cox proportional hazards model was used to calculate the hazard ratio (HR) and the 95% confidence interval (CI). Two models were used for the multivariable analysis. In Model 1, the AR was adjusted for all of the clinicopathologic factors. In Model 2, the AR was adjusted for factors that were significant in the univariable analysis. All of the statistical analyses were carried out using IBM SPSS Statistics, version 20.0 (IBM Corp., Armonk, NY, USA). All of the tests were two-sided and statistical significance was considered when the *p*-value was less than 0.05.

## 3. Results

### 3.1. Baseline Characteristics of the Study Subjects

The total number of subjects was 395 and their mean age was 53.3 ± 12.3 years (median, 51.0 years; range, 25–87 years). The study was conducted between July 1999 and April 2012. The mean follow-up duration for overall survival and disease-free survival was 120.8 ± 51.0 months (median, 121.0 months; range, 3–239 months) and 87.5 ± 50.5 months (median, 90.0 months; range, 1–216 months), respectively. The event numbers for death and any type of recurrence were 107 and 104, respectively. The subjects with low and high AR expression were 203 (51.4%) and 192 (48.6%), respectively (Table 1). The high AR expression group showed a higher proportion of patients with a tumor size ≤2 cm, a positive ER, a positive PR, a positive HER2, and a low histologic grade when compared with the low AR expression group. The high AR group showed higher proportions of the HRc(+)/HER2(−) and HRc(+)/HER2(+) subtypes and a lower proportion of the HRc(−)/HER2(−) subtype. The high AR expression group received more endocrine therapy. The baseline characteristics of the study subjects according to their AR status are summarized in Table 1.

### 3.2. Classification Method to Define Low vs. High Expression of AR

The high AR expression group showed superior disease-free survival when compared with the low AR group in all of the scoring systems including the proportion score (*p* = 0.004; cut-off value of 10%), the intensity score (*p* = 0.014; score 2, 3 vs. score 0, 1), another intensity score (*p* = 0.034; score 1, 2, 3 vs. score 0), and the Allred score (*p* = 0.042; score ≥3 vs. score 0, 2; Figure S2). We adopted the proportion score system with a cut-off value of 10% for further analyses because it revealed the most significant *p*-value. Both of the groups with a ≥50% proportion score and a 10%–50% proportion score showed better disease-free survival when compared with the ≤10% group, but no significant difference was observed between the groups of >50% and 10%–50% (Figure S3A). The group with an intensity score of 3 showed better disease-free survival when compared with the group with a score of 0 (*p* = 0.031), but no significant differences were observed between intensity score 3 and 2, and between intensity score 1 and 0 (Figure S3B).

### 3.3. Survival Analysis According to AR Status 

**Figure 1:**
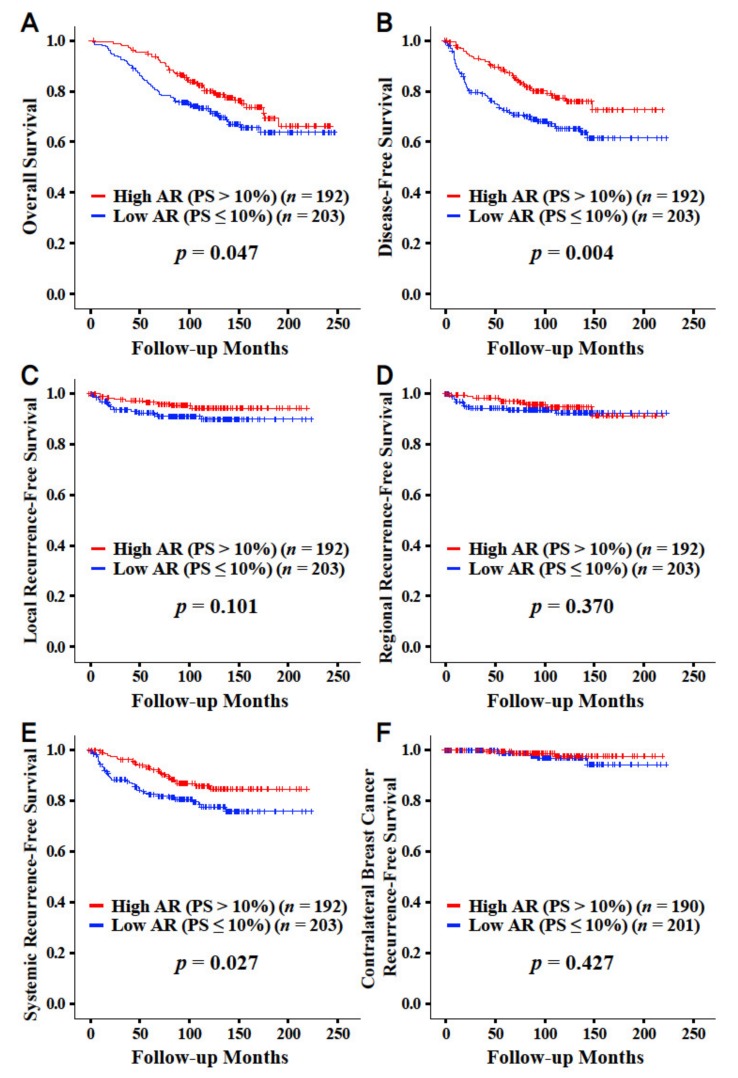
Overall survival and disease-free survival curves according to the proportion score with the cut-off value of 10% by immunohistochemistry regarding AR. Overall survival (**A**), disease-free survival (**B**), local recurrence-free survival (**C**), regional recurrence-free survival (**D**), systemic recurrence-free survival (**E**), and contralateral breast cancer recurrence-free survival (**F**). Abbreviation: AR, androgen receptor; PS, proportion score.

The high AR expression group showed both superior overall survival (*p* = 0.047) and disease-free survival (*p* = 0.004) when compared with the low AR expression group (Figure 1). The high AR expression group showed better systemic recurrence-free survival (*p* = 0.027; Figure 1E). No significant survival differences were observed when considering local, regional, and contralateral breast cancer recurrence. The detailed survival rates for overall survival and disease-free survival are described in Table S1.

### 3.4. Univariable and Multivariable Analyses 

The AR was a significant prognostic factor for overall survival in the univariable analysis (HR, 0.678; 95% CI, 0.462–0.997; *p* = 0.048; Table 2). In the multivariable analyses, the AR was an independent prognostic factor in both Model 1 (HR, 0.586; 95% CI, 0.381–0.901; *p* = 0.015) and Model 2 (HR, 0.619; 95% CI, 0.414–0.924; *p* = 0.019). The AR was a strong significant prognostic factor for disease-free survival in the univariable analysis (HR, 0.560; 95% CI, 0.377–0.834; *p* = 0.004; Table 3). In the multivariable analyses, the AR was also a strong independent prognostic factor in both Model 1 (HR, 0.430; 95% CI, 0.274–0.674; *p* < 0.001) and Model 2 (HR, 0.495; 95% CI, 0.328–0.745; *p* = 0.001).

### 3.5. Subgroup Survival Analyses According to the Expression Level of AR

**Figure 2:**
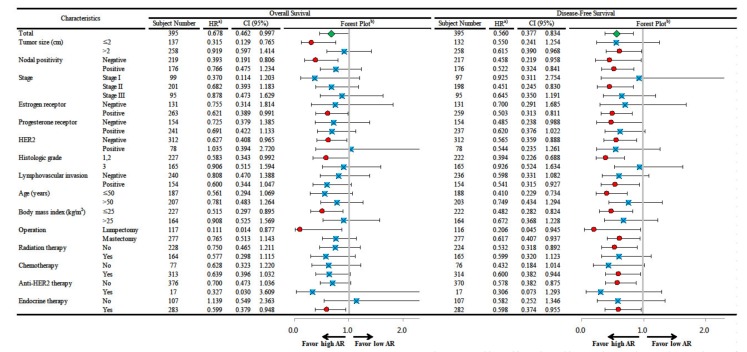
Subgroup analysis of the Cox proportional hazards model according to the expression level of AR (proportion score with the cut-off value of 10%) regarding overall survival and disease-free survival. Abbreviation: AR, androgen receptor; CI, confidence interval; HER2, human epidermal growth factor receptor 2; HR, hazard ratio. ^a)^ HRs are the relative risks of the high AR group (proportion score >10%) with reference to the low AR group (proportion score ≤10%) by Cox proportional hazards model. ^b)^ In the forest plot, an HR value of less than 1 favors the high AR group against the low AR group. The red circles mean statistical significance and the blue squares mean no statistical significance. The green diamond means the result of total subjects.

**Figure 3:**
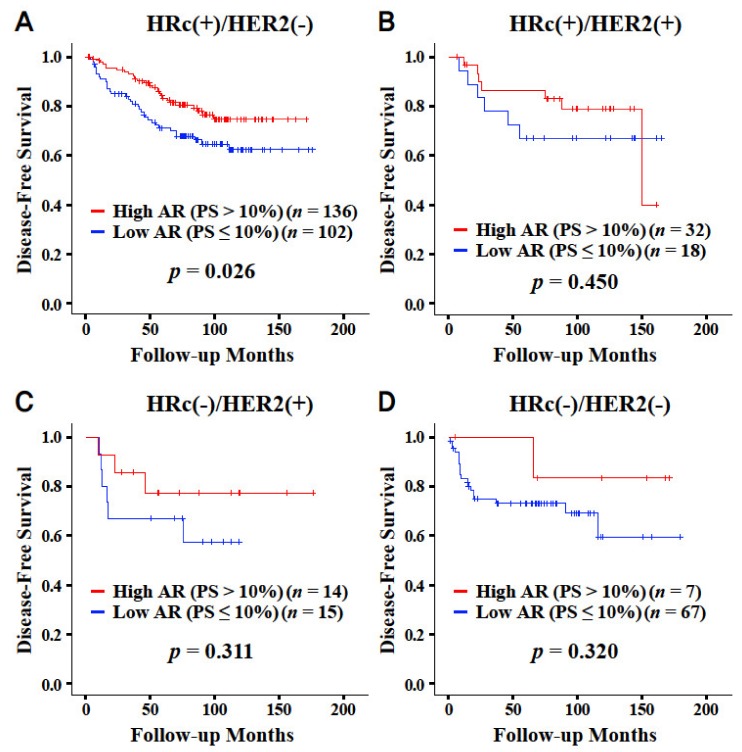
Disease-free survival curves according to the proportion score with the cut-off value of 10% by immunohistochemistry regarding AR. Curves in the subtype of HRc(+)/HER2(-) (**A**), HRc(+)/HER2(+) (**B**), HRc(-)/HER2(+) (**C**), and HRc(-)/HER2(-) (**D**), respectively. Abbreviation: AR, androgen receptor; HER2, human epidermal growth factor receptor 2; HRc, hormone receptor; PS, proportion score.

High AR expression was a significant favorable prognostic factor for overall survival in all of the subjects and subgroups with a tumor size of ≤2 cm, a negative node, a positive ER, a negative HER2, a histologic grade 1 or 2, a body mass index ≤25 kg/m^2^, a lumpectomy, and endocrine therapy (Figure 2). High AR expression was a significant favorable prognostic factor for disease-free survival in all of the subjects, and it was a significant favorable prognostic factor regardless of the nodal positivity and operation type. AR was also a significant prognostic factor in subgroups with a tumor size of >2 cm, stage II cancer, a positive ER, a negative PR, a negative HER2, a histologic grade 1 or 2, a positive lymphovascular invasion, age ≤50 years, a body mass index ≤25 kg/m^2^, no radiation therapy, chemotherapy, no anti-HER2 therapy, and endocrine therapy. High AR expression was a significant favorable prognostic factor for disease-free survival in the HRc(+)/HER2(−) breast cancer subgroup only (*p* = 0.026; Figure 3). AR was not a significant prognostic factor for overall survival in any of the breast cancer subtypes (Figure S4).

## 4. Discussion

This study investigated the prognostic role of AR in breast cancer using tissue microarrays from 395 patients with operable primary breast cancer. This study showed that the high AR expression group was significantly associated with better overall survival and better disease-free survival when compared with the low AR expression group. AR expression was a significant independent prognostic factor in terms of both overall survival and disease-free survival.

Previous studies have reported inconsistent results regarding the prognostic role of AR expression in breast cancer. The majority of the previous studies have reported a favorable prognostic effect for AR positivity [3,4,5,13,14,15,16]. Elebro et al. reported that positive AR status was a favorable prognostic marker for disease-free survival (*p* = 0.025) [13]. Aleskandarany et al. reported that AR expression is an independent prognostic marker for breast cancer-specific survival (HR, 0.71; 95% CI, 0.56–0.91; *p* = 0.007) [14]. The meta-analysis studies reported that AR-positive tumors were associated with both improved overall survival and disease-free survival [3,4]. However, some of the papers have insisted that there is no significant association between AR status and the prognosis of breast cancer [2,12,17]. Agrawal et al. reported that AR expression was not an independent prognostic factor for 10-year overall survival [17]. Asano et al. reported that AR had no association with overall survival or disease-free survival in patients who received neoadjuvant chemotherapy [12]. Kensler et al. analyzed data from the Breast International Group Trial 1–98, and reported that AR expression was not associated with breast cancer-free interval (HR, 1.07; 95% CI, 0.83–1.36; *p*  =  0.60) [2]. Furthermore, other studies have asserted the adverse prognostic effect of AR [6,18]. Zhang et al. reported that a high expression of AR in breast cancer patients was associated with shorter overall survival (103.18 vs. 84.71 months; *p* = 0.002) [18]. Jiang et al. reported that AR expression is correlated with decreased disease-free survival in triple-negative breast cancer (*p* = 0.014) [6]. In the current study, we showed the favorable prognostic effect of AR expression on both overall survival and disease-free survival in breast cancer. The prognostic impact of AR expression was more prominent in disease-free survival than in overall survival.

The proportion of tumors with positive AR expression in breast cancer patients varies widely, and mainly depends on the study population and the definition of AR positivity. The definition of AR status particularly has been highly inconsistent. A study that conducted a systemic review reported that AR-positive tumors accounted for 60.5% of all breast cancers, with a wide range of 28% to 84% [3]. Another meta-analysis study reported that AR positivity was 58.6% ranging from 12.8% to 78.7% [4]. Many studies defined AR positivity according to the proportion score with a cut-off value of 10% using immunohistochemistry, but other studies used cut-off values of 75%, 45%, 5%, 3% or 1% [3,4,6]. Ricciardelli et al. investigated to determine the optimal cut-off value for AR positivity as an independent predictor of breast cancer survival by ROC analysis with a comprehensive review of the literature [25]. They reported that prognostication was most robust with a high cut-off value of 78%, but not robust with lower (1%–10%) cut-off points. Some studies used other various parameters including the intensity score, the Allred score, the histoscore, the median value, and the receiver operating characteristic curve [3,4]. Other studies used radioimmunoassay methods instead of immunohistochemistry [3]. For the current study, we compared the performance of various cut-off values, which were derived from the proportion score, the intensity score, and the Allred score. We chose a cut-off value of 10% of the proportion score because it was best able to determine the survival difference. In this study, tumors with high AR expression accounted for 48.6% with a cut-off value of 10% using the proportion score. Differences in the study population and the definition of AR status across the literature could be the main factors causing the inconsistencies reported about the influence of the AR on the prognosis of breast cancer.

AR status is closely associated with certain clinicopathologic parameters. In this study, a high AR status was significantly associated with favorable clinicopathologic features such as small tumor size and low histologic grade. In particular, the AR status was most significantly associated with the ER/PR status. A significant association between AR and ER/PR has been reported in previous studies. Yu et al. reported that the AR expression was closely associated with the ER (*p* < 0.001) and the PR (*p* = 0.035), but not with the other conventional parameters such as T, N, stage, histologic grade, age, and HER2 [5]. Vera-Badillo et al. reported that AR-positive tumors were 74.8% and 31.8% in ER-positive and ER-negative tumors, respectively [3]. A close association between AR status and clinicopathologic features could partly explain the prognostic role of AR. A high AR status is associated with favorable clinicopathologic features and could be related to better survival. Furthermore, some previous studies have asserted that AR expression is different in ER-positive and ER-negative cancers and results in different clinical implications [15,19,20]. They reported that AR expression is associated with favorable clinicopathologic features and better clinical outcomes in ER-positive cancers, but not in ER-negative cancers.

AR expression varies across the breast cancer subtypes. Yu et al. reported that AR-positive cases accounted for 83.8%, 75.6%. 55.8%, and 39.0% in luminal A, luminal B, HER2 overexpressing, and basal breast cancer subtypes, respectively [5]. Jiang et al. reported that tumors with high AR expression were 52.3%, 34.4%, and 25.7% in luminal, HER2 positive, and triple-negative subtypes, respectively [6]. A review article reported that AR-positive tumors were 70%–95%, 50%–81%, and 12.5%–35% in ER-positive, HER2 positive, and triple-negative subtypes, respectively [7]. The prognostic role of AR also varies across breast cancer subtypes. Jiang et al. reported that AR positivity was associated with better disease-free survival in the luminal subtype (*p* < 0.001), but was associated with worse disease-free survival in the triple-negative subtype (*p* = 0.014) [6]. Asano et al. reported that positive AR expression had a worse prognosis for both overall survival (*p* = 0.002) and disease-free survival (*p* = 0.006) in triple-negative breast cancer patients who received neoadjuvant chemotherapy, but had no association in non-triple-negative breast cancers [12]. In the current study, the favorable effect of the high AR expression group was only valid in the HRc(+)/HER2(−) subtype for disease-free survival. This finding could be related to the results that suggest that the favorable effects of AR are only valid in the ER-positive and HER2 negative subgroups rather than the ER-negative and HER2 positive subgroups, respectively.

Among breast cancer subtypes, the triple-negative subtype is of major concern for researchers. Since Lehmann et al. reported that the luminal AR subtype, which was characterized by high expression of AR, accounted for a part of the triple-negative breast cancers [26], the AR signal pathway has attracted attention for its possibility as targeted therapy. The prognostic effect of AR in triple-negative breast cancer has been reported to be even more inconsistent than the effect in unselected breast cancers. Some studies have reported a favorable prognostic effect of AR in the triple-negative subtype [27,28,29]. Other studies have reported an adverse prognostic effect of AR in the triple-negative subtype [6,11,12]. Furthermore, some studies have reported no association between AR and the prognosis in triple-negative breast cancer [15,27,30,31].

The biological mechanism of AR in breast cancer varies according to its ER status. In ER-positive breast cancers, the AR and the ERα are thought to inhibit each other’s activity via multiple mechanisms for crosstalk between the two receptors [10]. A study reported that the AR potently inhibits the ERα activity which leads to the inhibition of breast cancer progression [8]. This mechanism could partly explain the favorable prognostic role of AR positivity in ER-positive breast cancers [3,15]. In ER-negative breast cancers, the existence of AR-dependent, and ER-independent tumor cell growth is related to the adverse prognostic effect of AR positivity, especially in the triple-negative subtype [6,11,12]. This mechanism proposed the potential for therapeutic strategies that target the AR to inhibit the androgen signaling pathway [32]. In particular, triple-negative breast cancer is the main subtype that is being targeted for the application of an anti-AR therapy, and is being actively investigated [33,34]. This study revealed that AR expression was a favorable prognostic factor in ER-positive breast cancer regarding both overall survival and disease-free survival. However, AR was not a prognostic factor in ER-negative tumors in the current study.

This study analyzed the detailed information of 395 primary breast cancer patients. The survival analyses and the long-term follow-up are significant strong points of this study. However, this study does have several limitations. First, this study analyzed a relatively small number of subjects, which probably weakened the statistical power. This is especially true for the subgroup analyses, which might have lost statistical significance due to the relatively small number of subjects. Second, this study adopted a cut-off value of 10% of the proportion score using immunohistochemistry to define the AR status. Because the definition of the AR status is crucial for AR studies, we hope that a standardized definition of AR status could be determined in the near future.

## 5. Conclusions

The high AR expression group was significantly associated with superior overall survival and disease-free survival when compared with the low AR expression group in breast cancer patients. The AR was a significant independent prognostic factor for both overall survival and disease-free survival. The high AR expression group was associated with favorable clinicopathologic features; especially with positive ER and PR. The prognostic impact of the AR was valid in the HRc(+)/HER2(−) subtype when considering disease-free survival. These findings as a whole suggest the clinical usefulness of AR as a prognostic marker of breast cancer in clinical settings.

## Figures and Tables

**Table 1:** Baseline characteristics of study subjects according to proportion score with the cut-off value of 10% by immunohistochemistry regarding AR.

Characteristics	All		Low AR (≤10%)		High AR (>10%)		*p* ^a^
	No.	%	No.	%	No.	%	
Total	395	100.00%	203	51.40%	192	48.60%	
Mean age (years)	53.3 ± 12.3		53.7 ± 12.7		52.8 ± 11.8		0.491
Tumor size (cm)							0.016
≤2	137	34.70%	59	29.10%	78	40.60%	
>2	258	65.30%	144	70.90%	114	59.40%	
Nodal positivity							0.131
Negative	219	55.40%	120	59.10%	99	51.60%	
Positive	176	44.60%	83	40.90%	93	48.40%	
Stage							0.279
Stage I	99	25.10%	44	21.70%	55	28.60%	
Stage II	201	50.90%	108	53.20%	93	48.40%	
Stage III	95	24.10%	51	25.10%	44	22.90%	
Estrogen receptor							<0.001
Negative	131	33.20%	105	51.70%	26	13.50%	
Positive	264	66.80%	98	48.30%	166	86.50%	
Progesterone receptor							<0.001
Negative	154	39.00%	101	49.80%	53	27.60%	
Positive	241	61.00%	102	50.20%	139	72.40%	
HER2							0.049
Negative	312	79.80%	169	83.70%	143	75.70%	
Positive	79	20.20%	33	16.30%	46	24.30%	
Unknown	4	1.00%	1	0.50%	3	1.60%	
Subtype							<0.001
HRc(+)/HER2(-)	238	60.30%	102	50.20%	136	70.80%	
HRc(+)/HER2(+)	50	12.70%	18	8.90%	32	16.70%	
HRc(-)/HER2(+)	29	7.30%	15	7.40%	14	7.30%	
HRc(-)/HER2(-)	74	18.70%	67	33.00%	7	3.60%	
Unknown	4	1.00%	1	0.50%	3	1.60%	
Histologic grade							<0.001
1, 2	227	57.90%	98	48.50%	129	67.90%	
3	165	42.10%	104	51.50%	61	32.10%	
Unknown	3	0.80%	1	0.50%	2	1.00%	
Lymphovascular invasion							0.529
Negative	240	60.90%	120	59.40%	120	62.50%	
Positive	154	39.10%	82	40.60%	72	37.50%	
Unknown	1	0.30%	1	0.50%	0	0.00%	
Age (years)							0.466
≤50	188	47.60%	93	45.80%	95	49.50%	
>50	207	52.40%	110	54.20%	97	50.50%	
Body mass index (kg/m^2^)							0.279
≤25	227	58.10%	112	55.40%	115	60.80%	
>25	164	41.90%	90	44.60%	74	39.20%	
Unknown	4	1.00%	1	0.50%	3	1.60%	
Operation							0.718
Lumpectomy	118	29.90%	59	29.10%	59	30.70%	
Mastectomy	277	70.10%	144	70.90%	133	69.30%	
Radiation therapy							0.937
No	228	58.00%	117	58.20%	111	57.80%	
Yes	165	42.00%	84	41.80%	81	42.20%	
Unknown	2	0.50%	2	1.00%	0	0.00%	
Chemotherapy							0.264
No	77	19.70%	35	17.50%	42	22.00%	
Yes	314	80.30%	165	82.50%	149	78.00%	
Unknown	4	1.00%	3	1.50%	1	0.50%	
Anti-HER2 therapy							0.401
No	376	95.70%	194	96.50%	182	94.80%	
Yes	17	4.30%	7	3.50%	10	5.20%	
Unknown	2	0.50%	2	1.00%	0	0.00%	
Endocrine therapy							<0.001
No	107	27.40%	74	36.80%	33	17.40%	
Yes	284	72.60%	127	63.20%	157	82.60%	
Unknown	4	1.00%	2	1.00%	2	1.00%	

Abbreviation: AR, androgen receptor; HER2, human epidermal growth factor receptor 2; HRc, hormone receptor. ^a^ *p*-value for mean age was calculated by *t*-test and all the other *p*-values were calculated by χ^2^ test.

**Table 2:** Univariable and multivariable analyses regarding overall survival.

Characteristics (All)	Univariable Analysis				Multivariable Analysis							
	HR	95% CI		*p*	Model 1 ^b^				Model 2 ^c^			
					HR	95% CI		*p*	HR	95% CI		*p*
AR, high vs. low ^a^	0.678	0.462	0.997	0.048	0.586	0.381	0.901	0.015	0.619	0.414	0.924	0.019
Tumor size (cm), >2 vs. ≤2	2.171	1.368	3.444	0.001	1.574	0.95	2.607	0.078	1.658	1.017	2.705	0.043
Nodal positivity, positive vs. negative	2.484	1.675	3.685	<0.001	1.811	1.116	2.94	0.016	1.905	1.192	3.045	0.007
Estrogen receptor, positive vs. negative	0.936	0.626	1.399	0.746	1.355	0.766	2.396	0.297				
Progesterone receptor, positive vs. negative	0.843	0.574	1.24	0.386	0.705	0.431	1.155	0.165				
HER2, positive vs. negative	0.789	0.476	1.31	0.36	0.762	0.448	1.298	0.318				
Histologic grade, 3 vs. 1, 2	1.376	0.942	2.01	0.099	1.196	0.784	1.825	0.406				
Lymphovascular invasion, positive vs. negative	1.85	1.262	2.712	0.002	1.519	0.957	2.409	0.076	1.475	0.946	2.302	0.087
Age (years), >50 vs. ≤50	1.797	1.211	2.667	0.004	1.252	0.804	1.949	0.32	1.374	0.909	2.077	0.132
Body mass index (kg/m^2^), >25 vs. ≤25	1.337	0.913	1.957	0.135	1.011	0.681	1.501	0.957				
Operation, mastectomy vs. lumpectomy	4.013	2.089	7.71	<0.001	3.085	1.511	6.298	0.002	2.68	1.371	5.238	0.004
Radiation therapy, yes vs. no	0.833	0.558	1.241	0.368	1.237	0.756	2.023	0.397				
Chemotherapy, yes vs. no	0.431	0.287	0.647	<0.001	0.34	0.21	0.551	<0.001	0.39	0.251	0.606	<0.001
Anti-HER2 therapy, yes vs. no	0.837	0.265	2.65	0.763	1	0.299	3.346	0.999				
Endocrine therapy, yes vs. no	0.781	0.516	1.183	0.243	0.901	0.503	1.615	0.727				

Abbreviation: AR, androgen receptor; CI, confidence interval; HER2, human epidermal growth factor receptor 2; HR, hazard ratio. ^a^ Expression of AR was classified into high and low using intensity score with the cut-off value of 10% by immunohistochemistry results. ^b^ AR factor was adjusted with all of 14 clinicopathologic factors including tumor size, nodal positivity, estrogen receptor, progesterone receptor, HER2, histologic grade, lymphovascular invasion, age, body mass index, operation, radiation therapy, chemotherapy, anti-HER2 therapy, and endocrine therapy. ^c^ AR factor was adjusted with 6 clinicopathologic factors, which were statistically significant by univariable analysis, including tumor size, nodal positivity, lymphovascular invasion, age, operation, and chemotherapy.

**Table 3:** Univariable and multivariable analyses regarding disease-free survival.

Characteristics (All)	Univariable Analysis				Multivariable Analysis							
	HR	95% CI		*p*	Model 1 ^b^				Model 2 ^c^			
					HR	95% CI		*p*	HR	95% CI		*p*
AR, high vs. low ^a^	0.56	0.377	0.834	0.004	0.43	0.274	0.674	<0.001	0.495	0.328	0.745	0.001
Tumor size (cm), >2 vs. ≤2	2.106	1.325	3.348	0.002	1.312	0.794	2.17	0.289	1.298	0.797	2.114	0.294
Nodal positivity, positive vs. negative	3.103	2.058	4.679	<0.001	2.308	1.397	3.813	0.001	2.227	1.373	3.61	0.001
Estrogen receptor, positive vs. negative	0.867	0.578	1.299	0.489	1.709	0.943	3.098	0.078				
Progesterone receptor, positive vs. negative	0.872	0.589	1.29	0.492	0.696	0.418	1.159	0.164				
HER2, positive vs. negative	1.054	0.659	1.688	0.826	0.78	0.453	1.345	0.371				
Histologic grade, 3 vs. 1, 2	1.411	0.961	2.073	0.079	1.316	0.857	2.022	0.21				
Lymphovascular invasion, positive vs. negative	2.306	1.561	3.406	<0.001	1.692	1.065	2.69	0.026	1.545	0.991	2.409	0.055
Age (years), >50 vs. ≤50	1.026	0.698	1.508	0.896	0.733	0.475	1.131	0.16				
Body mass index (kg/m^2^), >25 vs. ≤25	1.056	0.715	1.559	0.784	0.916	0.603	1.389	0.679				
Operation, mastectomy vs. lumpectomy	3.676	2.013	6.714	<0.001	3.162	1.627	6.146	0.001	3.037	1.643	5.613	<0.001
Radiation therapy, yes vs. no	0.891	0.6	1.323	0.568	1.024	0.636	1.648	0.923				
Chemotherapy, yes vs. no	0.828	0.516	1.327	0.432	0.555	0.319	0.966	0.037				
Anti-HER2 therapy, yes vs. no	2.279	1.105	4.7	0.026	3.207	1.378	7.466	0.007	2.284	1.096	4.759	0.027
Endocrine therapy, yes vs. no	0.699	0.46	1.061	0.092	0.762	0.409	1.419	0.392				

Abbreviation: AR, androgen receptor; CI, confidence interval; HER2, human epidermal growth factor receptor 2; HR, hazard ratio. ^a^ Expression of AR was classified into high and low using intensity score with the cut-off value of 10% by immunohistochemistry results. ^b^ AR factor was adjusted with all of 14 clinicopathologic factors including tumor size, nodal positivity, estrogen receptor, progesterone receptor, HER2, histologic grade, lymphovascular invasion, age, body mass index, operation, radiation therapy, chemotherapy, anti-HER2 therapy, and endocrine therapy. ^c^ AR factor was adjusted with 5 clinicopathologic factors, which were statistically significant by univariable analysis, including tumor size, nodal positivity, lymphovascular invasion, operation, and anti-HER2 therapy.

## Supplementary Materials

The following are available online at https://www.mdpi.com/2077-0383/9/4/1083/s1, Figure S1: Immunohistochemical staining results of AR. Proportion score of less than 10% (A), and more than 10% (B). Intensity score of 0 (C), 1 (D), 2 (E), and 3 (F), Figure S2: Disease-free survival curves according to expression level of AR by immunohistochemistry. Curves according to propensity score with the cut-off value of 10% (A), intensity score of 0, 1 vs. 2, 3 (B), intensity score of 0 vs. 1, 2, 3 (C), and Allred score of 0, 2 vs. more than 2 (D), Figure S3: Disease-free survival curves according to expression level of AR by immunohistochemistry. Curves according to proportion score (A) and intensity score (B), Figure S4: Overall survival curves according to proportion score with the cut-off value of 10% by immunohistochemistry regarding AR. Curves in the subtype of HRc(+)/HER2(-) (A), HRc(+)/HER2(+) (B), HRc(-)/HER2(+) (C), and HRc(-)/HER2(-) (D), respectively, Table S1: Detailed overall survival rates and disease-free survival rates according to expression level of AR (proportion score with the cut-off value of 10%).

## Abbreviations

AR	androgen receptor
CI	confidence interval
ER	estrogen receptor
HER2	human epidermal growth factor receptor 2
HR	hazard ratio
HRc	hormone receptor
PR	progesterone receptor
