# Supplementary material for: Influence of Androgen Receptor on the Prognosis of Breast Cancer
Source: J Clin Med. 2020 Apr 10;9(4):1083. doi: 10.3390/jcm9041083 (PMC7230528; doi:10.3390/jcm9041083)
Supplement: Supplementary file 1 [file jcm-09-01083-s001.pdf]

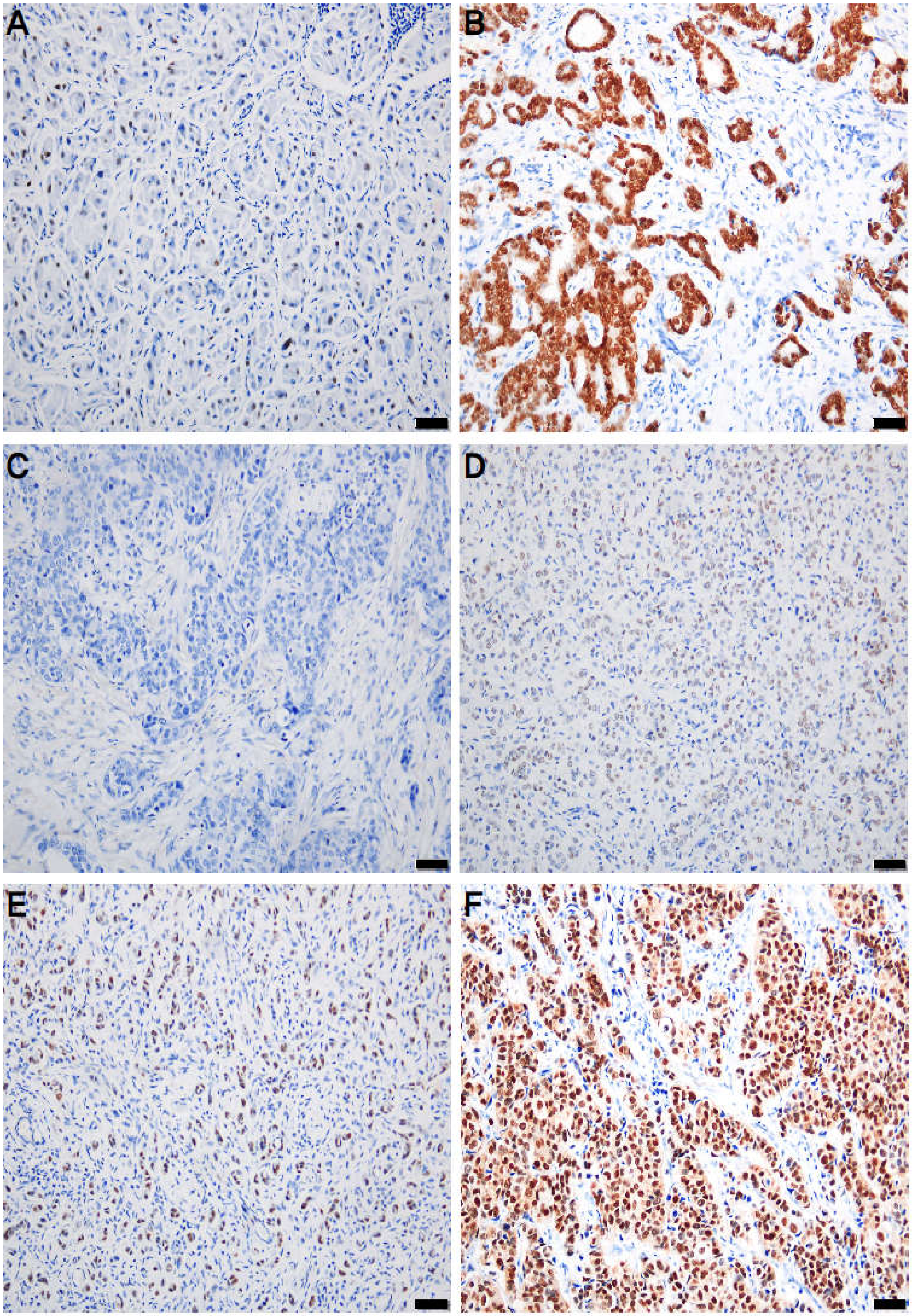

Figure S1. Immunohistochemical staining results of AR. Proportion score of less than 10% (**A**), and more than 10% (**B**). Intensity score of 0 (**C**), 1 (**D**), 2 (**E**), and 3 (**F**). Scale bar (bottom right), 50 μm. Abbreviation: AR, androgen receptor.

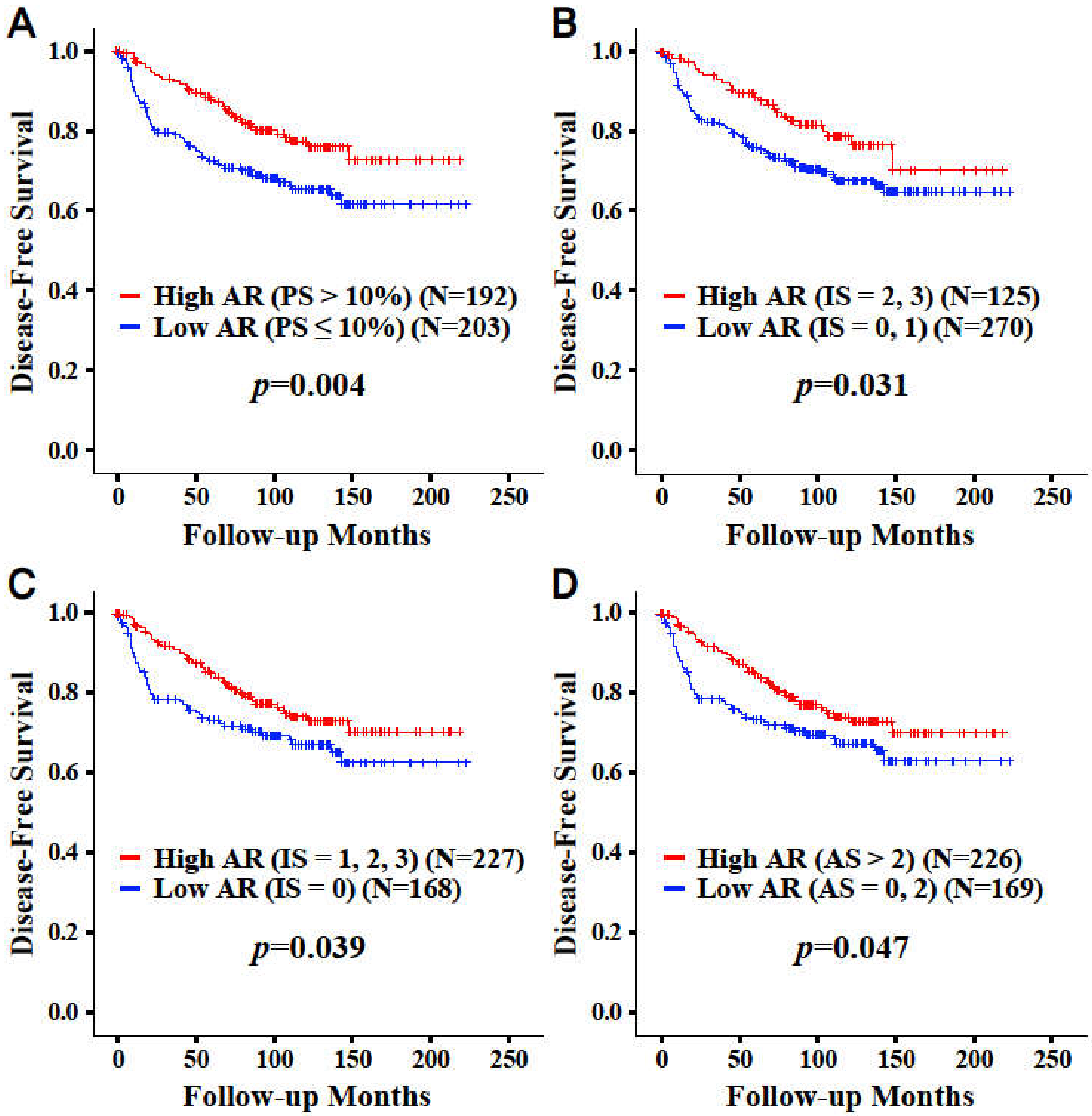

Figure S2. Disease-free survival curves according to expression level of AR by immunohistochemistry. Curves according to propensity score with the cut-off value of 10% (**A**), intensity score of 0, 1 vs. 2, 3 (**B**), intensity score of 0 vs. 1, 2, 3 (**C**), and Allred score of 0, 2 vs. more than 2 (**D**). Abbreviation: AR, androgen receptor; AS, Allred score, IS, intensity score; PS, proportion score.

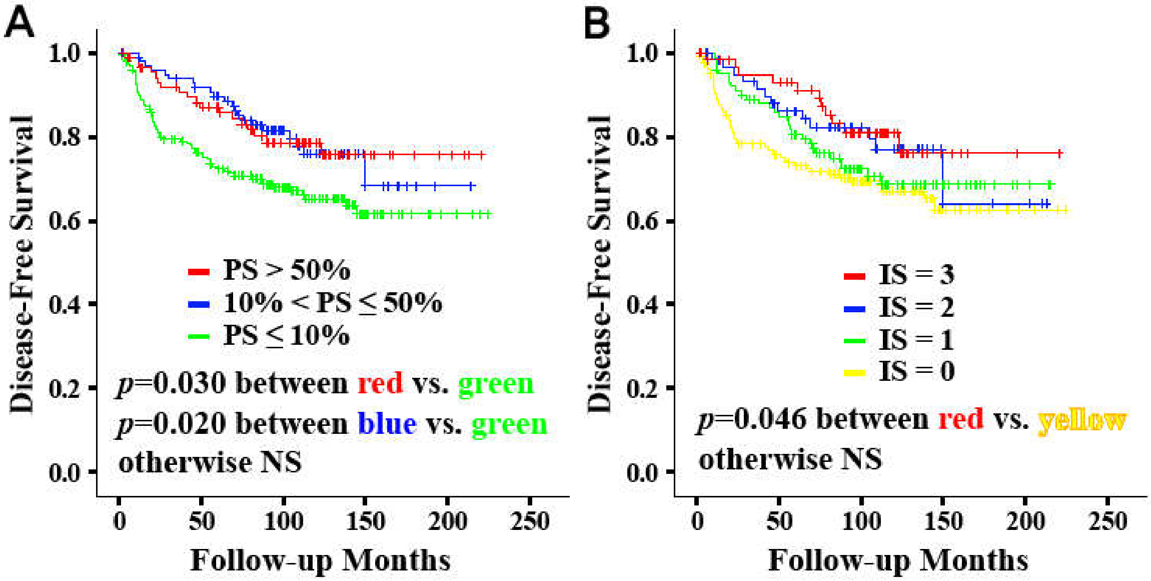

Figure S3. Disease-free survival curves according to expression level of AR by immunohistochemistry. Curves according to proportion score (**A**) and intensity score (**B**). Abbreviation: AR, androgen receptor; IS, intensity score; NS, not significant; PS, proportion score.

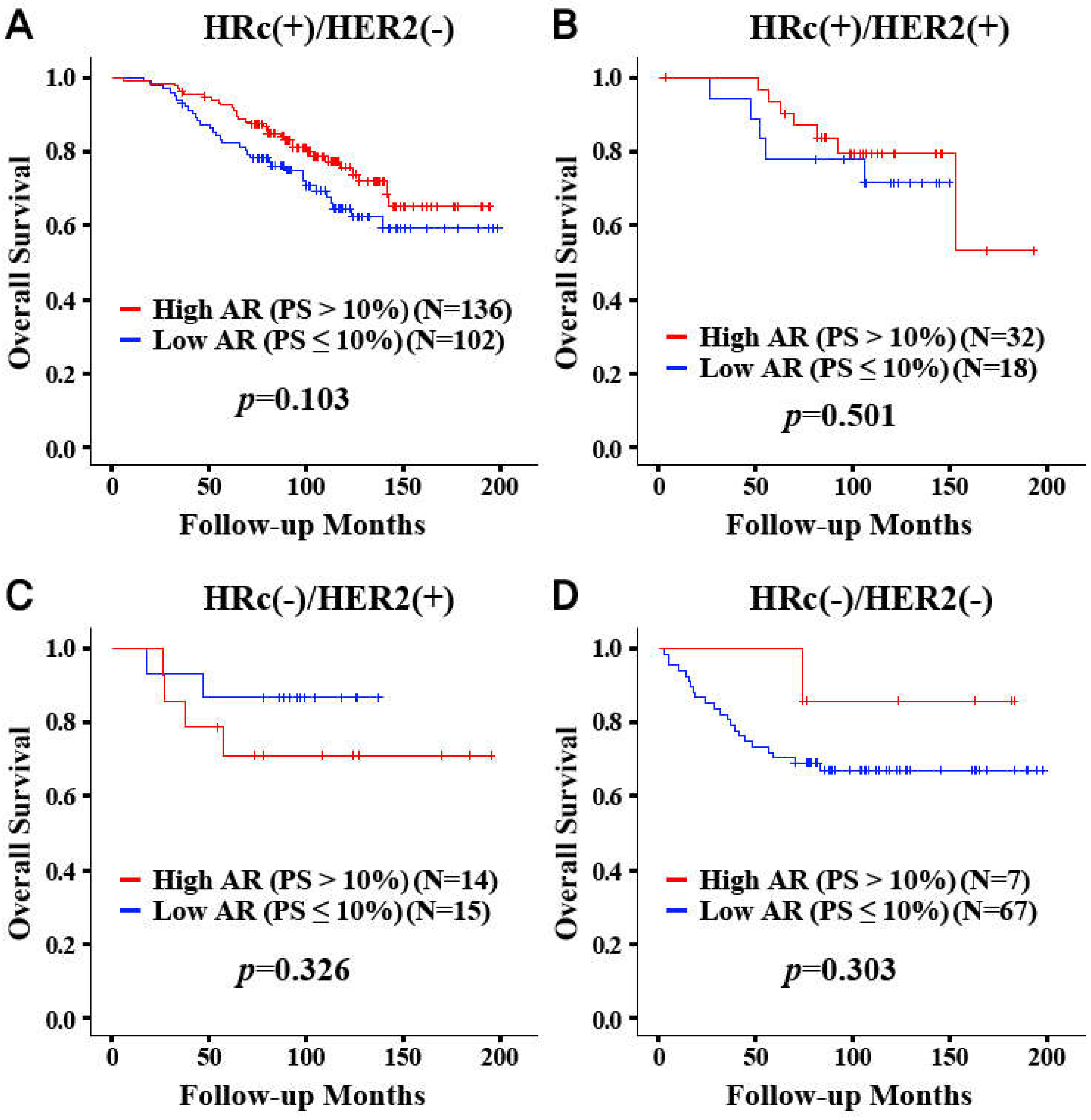

Figure S4. Overall survival curves according to proportion score with the cut-off value of 10% by immunohistochemistry regarding AR. Curves in the subtype of HRc(+)/HER2(-) (**A**), HRc(+)/HER2(+) (**B**), HRc(-)/HER2(+) (**C**), and HRc(-)/HER2(-) (**D**), respectively. Abbreviation: AR, androgen receptor; HER2, human epidermal growth factor receptor 2; HRc, hormone receptor; PS, proportion score.

Table S1. Detailed overall survival rates and disease-free survival rates according to expression level of AR (proportion score with the cut-off value of 10%).

**Overall Survival**									
	**Low AR**			**High AR**			**Total**		
**Follow up time (year)**	**Subject No**	**Event No [a)]**	**Rate**	**Subject No**	**Event No [a)]**	**Rate**	**Subject No**	**Event No [a)]**	**Rate**
5	203	36	82.20%	192	10	94.80%	395	46	88.30%
10	165	56	71.30%	178	35	80.30%	344	91	75.70%
15	97	63	63.90%	100	43	69.40%	198	106	66.60%
Last follow-up	19	63	63.90%	22	44	66.30%	42	107	65.00%
Last follow-up months	239 months			235 months			239 months		
**Disease-Free Survival**									
	**Low AR**			**High AR**			**Total**		
**Follow up time (year)**	**Subject No**	**Event No [a)]**	**Rate**	**Subject No**	**Event No [a)]**	**Rate**	**Subject No**	**Event No [a)]**	**Rate**
5	203	53	72.50%	192	22	87.90%	395	75	80.00%
10	127	63	65.40%	145	38	76.10%	273	101	70.60%
15	53	65	61.70%	48	39	72.70%	102	104	67.00%
Last follow-up	6	65	61.70%	8	39	72.70%	15	104	67.00%
Last follow-up months	216 months			212 months			216 months		

Abbreviation: AR, androgen receptor. [a)] Cumulative number of events.
